# Casomorphine-10 (CM-10) Peptide Orchestrates Circadian and Neurodevelopmental Gene Clusters via δ-Opioid Receptor Signaling: Insights from Transcriptome Analysis with δ-Opioid Receptor-Expressing HEK293 Cells

**DOI:** 10.3390/life15101636

**Published:** 2025-10-20

**Authors:** Moe Fukunaga, Shin Watanabe, Kanami Orihara, Naoyuki Yamamoto

**Affiliations:** 1 School of Life Science and Technology, Institute of Science Tokyo, Yokohama 226-8501, Kanagawa, Japan; 2 Department of Emergency and Disaster Medicine, Juntendo University, Hongo, Bunkyo-ku 113-8421, Tokyo, Japan; 3 Faculty of Medicine, Juntendo University, Hongo, Bunkyo-ku 113-8421, Tokyo, Japan

**Keywords:** opioid peptide, β-casomorphin-10, HEK293 expressing δ-opioid receptor, circadian rhythm, network analysis, developmental gene cluster

## Abstract

**Background:** β-casomorphin-10 (CM-10), a peptide fragment derived from milk casein with the sequence YPFPGPIPNS, has demonstrated notable anxiolytic activity in BALB/c mice. Yet, its cellular responses and mechanistic pathways remain largely uncharacterized. **Methods:** We performed RNA-seq analysis to profile gene expression changes in δ-opioid receptor-expressing HEK293 cells (DOR-HEK), comparing CM-10-treated and untreated conditions. **Results:** CM-10 exposure led to differential expression of 1714 genes in DOR-HEK cells, with 34 upregulated (>1.4-fold) (1.9%) and 1680 downregulated (<0.71-fold) (98.1%), based on a predicted *p*-value threshold of <0.05. Notably, we identified 10 clusters that were associated with reduced cyclic AMP (cAMP) in DOR-HEK cells following CM-10 treatment. These clusters particularly involved genes related to regulatory subunits of cAMP-dependent protein kinases, such as *PRKAR2A*, cAMP-responsive element-binding pathway, circadian rhythms, such as *CLOCK*, *ARNT1*, *CRY2*, *PER1*, and *PER2*, and anxiety and depression, such as *NOTCH1*, *NOTCH2* and *ANK2*. A network with these selected genes was confirmed by STRING analysis. **Conclusions:** These findings indicate that CM-10 may activate DOR-mediated signaling by suppressing cAMP levels, implicating a distinct molecular cascade in HEK293 cells.

## 1. Introduction

Various food proteins can release short, hidden biological peptides via proteolytic actions. Proteolytic hydrolysis is used to generate many biologically active peptides, including antihypertensive [1], antioxidant [2], immunomodulatory [3], antimicrobial [4], and opioid peptides [5], from various food proteins for application in functional food materials [6,7]. Food protein-derived opioid peptides exhibiting agonistic effects by binding to specific receptors hold great potential for the treatment of anxiety, hypertension, and stress [8]. Consequently, exogenous opioid peptides with biological functions similar to those of endogenous opioid peptides have been isolated via the enzymatic hydrolysis of food proteins [9,10].

The four endogenous opioid systems consist of enkephalins, β-endorphins, dynorphins, and nociception, with four corresponding receptors—µ-opioid receptor (MOR), δ-opioid receptor (DOR), κ-opioid receptor, and nociceptin-opioid receptor [11,12,13]—and are widely distributed across neuraxis and pain pathways [11]. Additionally, they also control various different functions, involving anxiety, stress responses, depression, reward/aversion behavioral responses, gastrointestinal transit, and neuroendocrine and immune functions [14,15,16]. Endogenous enkephalin exposure in DOR-expressing HEK293 cells results in receptor-dependent STAT5B tyrosine phosphorylation and transcriptional activation [17].

The exogenous opioid peptide, Tyr–Pro–Phe–Pro–Gly–Pro–Ile, with morphine-like activity was first isolated from casein hydrolysate as β-casomorphin-7 [18]. Some opioid peptides have also been isolated from the hydrolysates of the soy protein β-conglycinin [19]. Exorphine A5 and A4, the opioid peptides Gly–Tyr–Tyr–Pro–Thr and Gly–Tyr–Tyr–Pro, respectively, have been isolated from wheat gluten [20]. In our previous study, we identified an opioid peptide β-casomorphin-10 (CM-10) comprising the YPFPGPIPNS sequence (β-casein 60–69) from the casein hydrolysate using *Aspergillus oryzae* protease [21] in DOR-expressing HEK293 (DOR-HEK293) cells [22]. Notably, CM-10 interacts with DOR and MOR and docks into the optimized DOR in the simulation analysis. A significant anxiolytic effect was observed in BALB/c mice in an elevated plus maze study following oral but not intravenous CM-10 administration [21].

Delta agonists promote anxiolytic and anti-depressant effects by interacting with DOR [23]. MOR agonists also exerted anxiolytic effects in the elevated plus maze tests [24]. However, mechanisms of action of opioid peptides after interaction with DOR and cAMP reduction remain unknown. Moreover, there is no report to suggest the possible potential of opioid peptides from a wide range of transcriptome analyses after peptide treatment. Notably, the analgesic activity of curcumin has been elucidated via transcriptome analysis based on mRNA sequencing in human T98G neuroglial cells, followed by an interactive pathway analysis of regulated genes [25].

To detect specific agonistic signals from host cells after CM-10 treatment, DOR-HEK cells were used for the transcriptome analysis. The present study aimed to clarify the host responses in DOR-HEK cells following CM-10 exposure using wide transcriptome and network analyses.

## 2. Materials and Methods

### 2.1. Cell Experiment

#### 2.1.1. Reagents and Materials

Dulbecco’s modified Eagle’s medium (DMEM) was purchased from Nacalai Tesque, Inc. (Kyoto, Japan). Fetal bovine serum (FBS) was obtained from Life Technologies Japan, Ltd. (Tokyo, Japan). Geneticin-selective antibiotic was purchased from Life Technologies Japan, Ltd. CM-10 was synthesized by GenScript (Nanjing, China).

#### 2.1.2. HEK293 and DOR-HEK Cell Culturing

HEK and HEK293 cells stably expressing human Halo Tag-DOR pGS22F cyclic AMP (cAMP)/hygro (DOR-HEK293) were kindly provided by Prof. Uezono (National Cancer Center Japan Research Institute, Tokyo, Japan) [22]. HEK and DOR-HEK293 cells were cultured in DMEM containing 10% FBS and 1% penicillin–streptomycin (Wako, Japan) and hygromycin B (Wako, Japan; 100 μg/mL) at 37 °C and 5% CO_2_. Geneticin-selective antibiotic (700 μg/mL) (Life Technologies Japan, Ltd., Tokyo, Japan) was added to DOR-HEK293 cells. Both cell lines were stained with an anti-DOR antibody.

#### 2.1.3. DOR Detection on HEK293 Cells

DOR-HEK293 cells cultured in DMEM containing 10% FBS and 1% penicillin–streptomycin and hygromycin B were fixed using the BD Cytofix/Cytoperm™ Solution Kit (BD Biosciences, San Diego, CA, USA) and permeabilized with 0.5% Triton in phosphate-buffered saline (PBS) (pH 7.4) to confirm DOR expression and localization on the surface of HEK293 cells. HEK293 cells were also cultured in similar DMEM and fixed with the BD Cytofix/Cytoperm™ Solution Kit. The fixed DOR-HEK293 and HEK293 cells were then incubated with a rabbit anti-DOR antibody (GeneTex, Irvine, CA, USA) after dilution with 1% casein in PBS (1/1000) and stained with a secondary antibody (Cy3-conjugated anti-rabbit IgG, Rockland Inc., PA, USA).

### 2.2. Bioinformatic Analysis

#### 2.2.1. mRNA Preparation and Sequencing

Quick agonistic opioid activity was observed in DOR-HEK cells after exposure to CM-10 in a previous study [21]. Therefore, DOR-HEK293 cells were cultured in DMEM with or without CM-10 treatment for 1 h to understand rapid cell responses. Gene expression with and without CM-10 treatment was compared using RNA sequencing (RNA-seq). DOR-HEK293 cells were seeded at a density of 5.0 × 10^4^ cells/well in a 24-well culture plate and cultured for 4 days. After removing the culture supernatant, fresh DMEM containing 10% FBS, 1% penicillin–streptomycin and hygromycin B (100 μg/mL) was added (control sample). For the CM-10 sample, CM-10 was added to the control group (1.54 mM) because 1 mM CM-10 showed opioid activity against DOR-HEK293 cells in a previous study [21]. Both samples were incubated at 37 °C and 5% CO_2_ for 1 h. The cells were then washed with PBS and total RNA was extracted. The total RNA concentration was calculated using Quant-IT RiboGreen (Thermo Fisher Scientific, Waltham, MA, USA). Samples with A260/A280 ratios between 1.8 and 2.2 were used for subsequent analysis. For RNA-seq, equal amounts of RNA from triplicate samples from the CM-10 group were mixed and compared with those from the control group. The first step in the workflow involved the purification of poly A-containing mRNA molecules using poly T-attached magnetic beads. The cleaved RNA fragments were copied into first-strand cDNA using SuperScript II reverse transcriptase (Thermo Fisher Scientific) and random primers. This was followed by the synthesis of second-strand cDNA using DNA Polymerase I, RNase H, and dUTP. These cDNA fragments were then subjected to an end repair process, the addition of a single ‘A’ base, and ligation of the adapters. The products were purified and enriched using PCR to generate a final cDNA library. To assess the integrity of the total RNA, samples were run on a TapeStation RNA ScreenTape (Agilent, Tokyo, Japan). A library was independently prepared with 1 μg of total RNA from each sample using the Illumina TruSeq Stranded mRNA Sample Prep Kit (Illumina Inc., San Diego, CA, USA). Libraries were quantified using KAPA Library Quantification kits for Illumina Sequencing platforms according to the qPCR Quantification Protocol Guide (KAPA BIOSYSTEMS, London, UK) and qualified using a TapeStation D1000 ScreenTape (Agilent Technologies, Tokyo, Japan). The indexed libraries were then subjected to Illumina NovaSeq (Illumina, Inc., San Diego, CA, USA), and paired-end (2 × 100 bp) sequencing was performed by Macrogen Inc. (Tokyo, Japan).

#### 2.2.2. mRNA-Seq Data Set Processing

We preprocessed the raw reads from the sequencer to remove low-quality and adapter sequences before analysis and aligned the processed reads to *Homo sapiens (hg38)* using HISAT v2.1.0 [26]. HISAT uses two types of indices for alignment (a global whole-genome index and tens of thousands of small local indices). These two types of indexes were constructed using the Burrows–Wheeler transform (BWT)/graph FM index (GFM), as in Bowtie 2. Owing to the use of these efficient data structures and algorithms, HISAT generates spliced alignments several times faster than Bowtie and BWA, which are widely used methods. The reference genome sequence of *H. sapiens (hg38)* and annotation data were downloaded from the UCSC Table Browser (https://genome.ucsc.edu). Transcript assembly and abundance estimation were performed using StringTie [27,28]. After alignment, StringTie v2.1.3b was used to assemble the aligned reads into transcripts and estimate their abundance. It provides relative abundance estimates as Read Count values of transcripts and genes expressed in each sample. Transcript assembly of known, novel, and alternative splicing transcripts was performed using StringTie v2.1.3b. Based on the results, the expression abundance of transcripts and genes was calculated as the read count or Fragments Per Kilobase of exon per million fragments mapped (FPKM) value per sample. Expression profiles were used to perform additional analyses, such as Differentially Expressed Genes (DEGs). In groups under different conditions, DEGs or transcripts were filtered using statistical hypothesis testing. DEGs with fold changes of >1.4 and <0.71 (1/1.4) and predicted *p*-value using EdgeR Version 4.6.3. [29] tool of less than 0.5 were selected. After the detection of DEGs, gene ontology (GO), cluster analysis, and molecular interactions between the upregulated and downregulated genes were visualized using STRING analysis (https://string-db.org/).

#### 2.2.3. qPCR of the Selected Genes

After network analysis, gene expressions of various key genes in the selected clusters were quantified. The gene expressions of triplicate samples in each group with the specific primers are listed in Table 1. Extracted RNA was reverse-transcribed (RT) into complementary DNA (cDNA) using a quantitative polymerase chain reaction (qPCR) RT master mix following the manufacturer’s instructions. The resulting cDNA was diluted to 10 ng/μL with nuclease-free water and mixed with specific primers and the SYBR qPCR mix. Mixtures were incubated in a StepOne Real-Time PCR system (Applied Biosystems, Waltham, MA, USA) following the manufacturer’s instructions. β-actin was used as a reference to normalize mRNA expression because the gene was stably expressed in DOR-HEK293 cells with or without CM-10 treatment.

#### 2.2.4. Statistical Analysis

For RNA-seq analysis, DEGs with fold changes of >1.4 and <0.71 (1/1.4) and predicted *p*-value using EdgeR [29] of less than 0.5 were selected. All qPCR analyses were performed on three samples from one group each of the CM-10 and control groups. The normality of the data was tested using the Shapiro–Wilk test. Non-parametric tests were used for analysis, and two-group comparisons were performed using the Mann–Whitney U-test with Prism software Version 10 (Prism Systems, Inc., Mobile, AL, USA). Statistical significance detected in triplicate samples was set at *p* < 0.05.

## 3. Results

### 3.1. DOR Expression on DOR-HEK293 Cells

**Figure 1:**
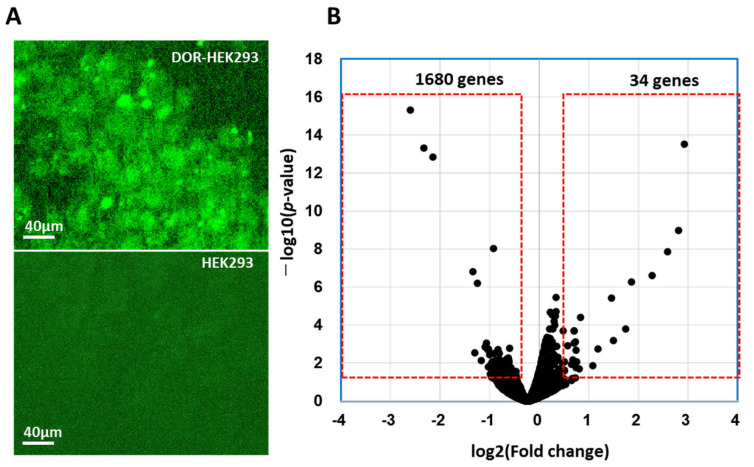
δ-opioid receptor (DOR)-expressing HEK293 cells and HEK293 cells stained with anti-DOR antibody and followed with FITC-conjugated anti-mouse IgG (**A**). Volcano plot of identified differentially expressed genes (DEGs) in DOR-HEK293 cells after 1 h treatment with CM-10 (**B**). Red boxes show changed gene expression over 1.4-time higher and less than 0.71 with *p*-value predicted by EdgeR [29] of less than 0.05.

In a previous study, CM-10 was isolated from casein hydrolysate by selecting peptides with agonistic effects on DOR-HEK293 cells by monitoring the inhibited adenylate cyclase activity [30] in the GloSensor cAMP system [31]. Notably, CM-10 specifically interacted with DOR and showed stable docking with DOR in a simulation analysis [21]. Therefore, we evaluated gene expression in DOR-HEK293 cells following the CM-10 interaction with DOR. Immunostaining with an anti-DOR antibody followed by an FITC-anti-rabbit antibody confirmed DOR expression in DOR-HEK293 cells (Figure 1A). Notably, specific expression of DOR was observed upon DOR gene introduction into HEK293 cells, whereas no significant signal was observed in HEK293 cells without DOR introduction (Figure 1A).

### 3.2. DEGs in DOR-HEK293 Cells Following CM-10 Treatment

Opioid receptors belong to the family of G-protein-coupled receptors, which inhibit adenylate cyclase upon activation [32,33]. Among the reported opioid receptors, DOR is associated with chronic pain stress [34], and its agonists are effective in treating migraine [34] by positively modulating emotional tone. We performed transcriptome analysis by an RNA-seq method to understand the mechanism of action underlying adenylate cyclase inhibition via the interaction between the opioid peptide CM-10 and DOR in HEK293 cells. Accordingly, we sought to identify DEGs in CM-10-treated and non-treated DOR-HEK293 cells (CM-10 and control groups). Notably, 1680 DEGs exhibiting >1.4- and <0.71-fold changes were identified between the CM-10 and control groups. Among the detected DEGs, 1680 (98.1%) genes were downregulated and 34 (1.9%) genes were upregulated following CM-10 treatment (Supplementary Tables S1 and S2, respectively). The volcano plot also revealed differential mRNA expression between the CM-10 and control groups, with the downregulation of most of the repressive genes in the CM-10 group (Figure 1B).

### 3.3. Ontology Analysis

**Figure 2:**
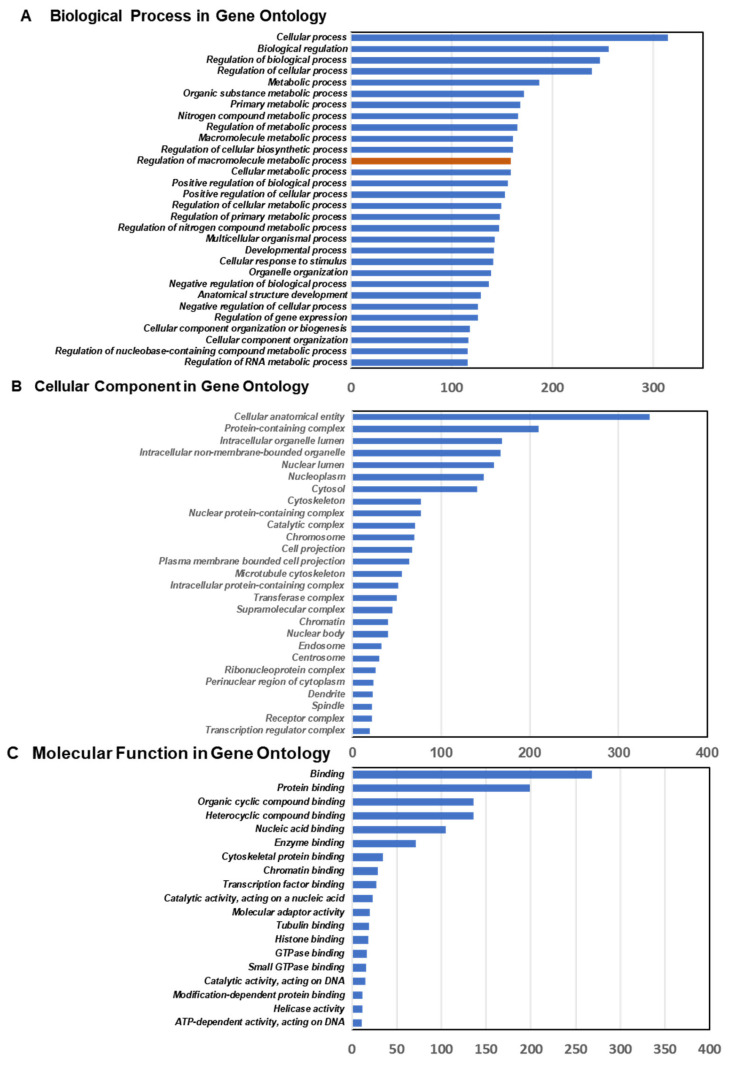
Ontology analysis for biological process (**A**), cellular component (**B**), and molecular function (**C**). Regulation of macromolecule metabolic process involving key circadian rhythms in biological processes was marked in orange.

To understand the biological functions of CM-10 based on the identified DEGs in DOR-HEK293 cells, we performed the Gene Ontology (GO) annotation for DEGs via a comparative analysis of CM-10-treated and non-treated DOR-HEK293 cells and molecular interactions among the detected DEGs. Figure 2 presents the DEGs and GO categories with the lowest *p*-values for biological processes (Figure 2A), cellular components (Figure 2B), and molecular functions (Figure 2C). Notably, “Regulation of macromolecule metabolic process” in biological processes includes circadian rhythm-linked genes, such as *CLOCK*, *PER2*, *ARNT*, and *CREBBP*. For biological processes, the top 10 clusters were cellular processes, biological regulation, regulation of biological processes, regulation of cellular processes, metabolic processes, organic substance metabolic processes, primary metabolic processes, nitrogen compound metabolic processes, regulation of metabolic processes, and macromolecule metabolic processes (Figure 2A, Supplementary Table S3). For cellular components, the top 10 clusters were cellular anatomical entities, intracellular organelle lumen, nuclear lumen, cytosol, nuclear protein-containing complex, chromosome, plasma membrane-bound cell projection, intracellular protein-containing complex, supramolecular complex, and nuclear body (Figure 2B, Supplementary Table S4). For molecular functions, the top 10 clusters were binding, protein binding, organic cyclic compound binding, heterocyclic compound binding, nucleic acid binding, enzyme binding, cytoskeletal protein binding, chromatin binding, transcription factor binding, and catalytic activity, acting on a nucleic acid (Figure 2C, Supplementary Table S5).

### 3.4. Network Analysis of DEGs

**Figure 3:**
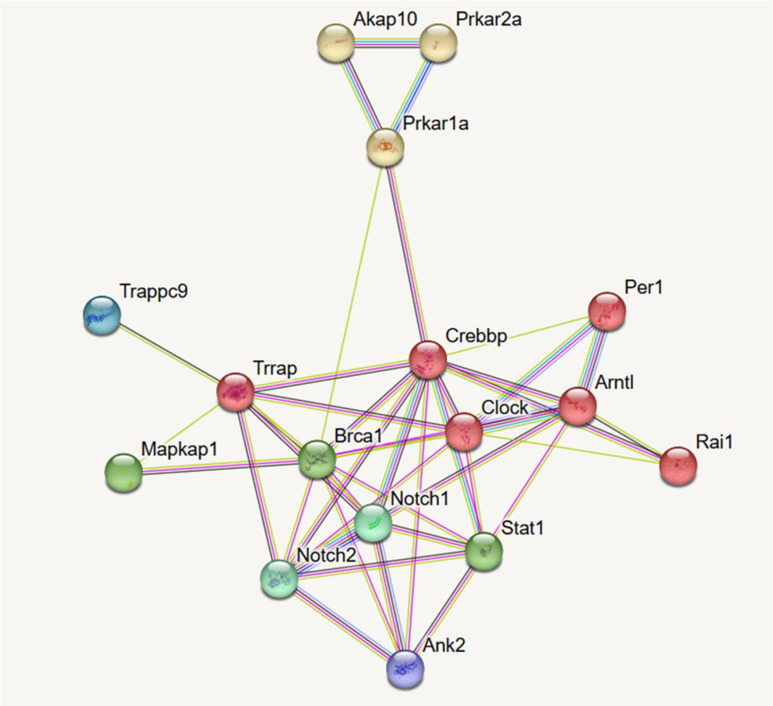
Predicted DOR agonistic signaling networks in DOR-HEK293 cells after 1 h treatment with CM-10. Fourteen genes with suggested involvement in cAMP-dependent protein kinases, transcriptional regulators in response to cAMP, circadian rhythm, stress and depression are shown in Table 2 and were applied for network analysis by STRING. Red: circadian rhythm, Green: regulation of transcription of Notch receptor target, Yellow: PKA activation in glucagon signalling.

To understand the possible pathways activated in DOR-HEK293 cells following CM-10 treatment, credible 10 network clusters were predicted from the identified DEGs (Supplementary Tables S1 and S2) using STRING analysis (https://string-db.org/). Network analysis suggested stronger connections between Clusters 9, 10, 6, and 5 (Figure S1). Among the predicted 10 major clusters, Cluster 1 included the main network of centrosomal protein genes, such as *CEP135*, *CEP152*, and *CEP295* (Table 2, Figure S1). CEP135 affects various cellular processes, including the cell cycle [35]. In Cluster 2, the main network included genes such as *UTP15*, *UTP20*, *RRP1*, *RRP12*, and *RRP1B*; however, the links with other clusters were not clear. Cluster 3 also showed no clear associations with the other clusters involved in protein trafficking for autophagy, such as *TRAPPC10*, *TRAPPC11*, *TRAPPC2L*, *TRAPPC6B*, *TRAPPC8*, and *TRAPPC9* [36], and exocyst complex components involved in cell cycle progression [37], morphogenesis, migration, and autophagy, such as *EXOC1*, *EXOC4*, *EXOC6*, and *EXOC6B* [37]. Cluster 4 was the coiled-coil domain cluster containing *CCDC66* [38] and *CCDC97*, splicing factors *SF3B1*, *PRPF3*, and *PRPF4B*, and protein phosphorylation factors *PPP2R1B* and *PPP2R5E*. Cluster 5 included *NOTCH1* and *NOTCH2*, which may be associated with depression and anxiety-like behaviors [39]. Cluster 5 also included *ANK2*, which encodes the AnkB required for connections in the central nervous system [40]. Interestingly, Cluster 6 mainly included various genes involved in the circadian rhythm, such as *CLOCK*, *PER1*, *RAI1*, *NR1D1*, *NR1D2*, *ARNTL*, *CIPC*, and *AHR*, and histone modifications, such as *KDM6B*, *EZH2*, and *JARDID2*. Cluster 7 included various epigenetic transcriptional regulator genes, such as *TRRAP*, *TELO2*, *BRD1*, *YEATS2*, *KAT6A*, and *MAX*, and transcriptional regulators, such as *BTAF1*, *INO80*, and *SRCAP*. Cluster 8 included nucleocytoplasmic transport genes, such as *NUP210*, *NUP58*, and *NUP155*, and regulators of TGF-beta/activin signaling, such as *VPS33A*, *VPS39*, and *VPS18*. Cluster 9 involved genes related to cAMP-dependent protein kinases probably involved in the initial step of cAMP signaling, such as *PRKAR1A*, *PRKAR2A*, and *AKAP10* [40,41], and GPCR coupled protein modification, such as *TTC21B* and *TULP3*. Cluster 10 involved transcriptional regulators, such as *CREBBP* and *BRCA1* [42], which are associated with cAMP-dependent protein kinases reported in Cluster 9 [43]. The selected 14 genes that formed the network (Figure 3) are probably involved in circadian rhythm metabolism, such as *TRAPP*, *TRAPPC9*, *ANK2*, *NOTCH1*, *NOTCH2*, *ARNTL*, *CLOCK*, *PER1*, *RAI*, *AKAP10*, *PRKAR1A*, *PRKAR2A*, *BRCA1*, and *CREBBP*.

### 3.5. Gene Expression Validation Using qPCR

**Figure 4:**
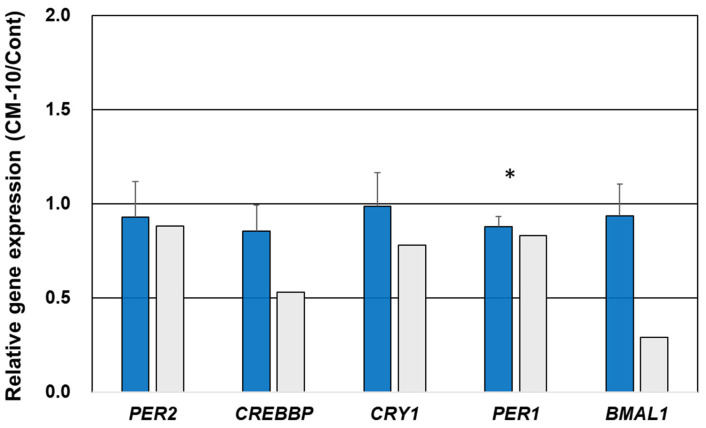
Comparative analysis of gene expression by RNA-seq and qPCR. Fold change in gene expression (CM-10/control) by qPCR (blue) and RNA-seq (light gray). Data for qPCR are represented as mean ± SD (*n* = 3/group Mann–Whitney U-test). Significant differences from control group analyzed by * *p* < 0.05.

Various clusters were identified using the network analysis (Figure S1, Table 2). Among the observed 10 clusters, the most credible network Clusters 5, 6, 9, and 10 with higher connections (blue clusters) were selected for validation analysis. Circadian rhythm-linked genes observed in Cluster 6 and transcriptional regulator genes observed in Cluster 10 were selected to validate gene expression using qPCR. All selected genes exhibited lower expression in the CM-10-treated group than in the control group, similar to the results observed using RNA-seq, except for *CRY2* (Figure 4). Notably, the expression of the other five genes, *PER2*, *CREBBP*, *CRY1*, *PER1*, and *PER3*, also showed trends similar to those obtained using RNA-seq (Figure 4). Significant downregulation of *PER1* was observed using qPCR, along with the downregulation of *PER2*, *CREBBP*, *PER1*, *CRY2*, *PER3*, and *CREBBP* following CM-10 treatment (Figure 4).

## 4. Discussion

Various opioid peptides have been isolated from food protein hydrolysates prepared using proteolytic enzymes [44,45] and synthetic methods [46]. Among them, some opioid peptides have shown anxiolytic effects in the elevated plus maze test [47,48]. However, the mechanism of in vivo action of exogenous opioid peptides [19,49] remains unknown, probably owing to their mild effects. In this study, we attempted to explore the effects of CM-10 on DOR-expressing cells and their gene expression in vivo. To evaluate the agonistic potential of CM-10, DOR-HEK293 cells were subjected to transcriptome analysis, followed by network analysis, with or without CM-10 treatment. Among the identified 1714 DEGs, most genes (98%) were significantly downregulated following CM-10 treatment. Furthermore, we also predicted the major 10 agonistic pathways, as illustrated in Figure S1. The most credible network Clusters 5, 6, 9, and 10 suggested that the CM-10 and DOR interaction may affect the cAMP-dependent kinase pathway, transcriptional regulation, and circadian rhythm regulation. Here, we report, for the first time, the potential of the exogenic opioid peptide CM-10 on agonistic pathways in DOR-HEK293 cells using RNA-seq.

DOR is a GPCR that is co-expressed with G proteins in various tissues [50] and promotes downstream signaling in response to agonistic opioids [51]. Interaction between agonistic opioids and DOR can reduce cAMP via adenyl cyclase, which catalyzes cAMP synthesis from ATP in target cells. Subsequently, cAMP binds to the regulatory subunits of cAMP-dependent protein kinase A (PKA product), thereby releasing catalytic subunits [52]. In the present study, *PRKAR1A* and *PRKAR2A* genes, which encode two regulatory subunits of a heterotetramer kinase (*RKAR1A*, *PRKAR2A*, *PRKAR1B*, and *PRKAR2B*), were downregulated following CM-10 treatment (Cluster 9 in Figure S1). Furthermore, *AKAP10*, a member of the cAMP-dependent protein subunit of the PKA holoenzyme family, was also downregulated following CM-10 treatment. Therefore, the downregulation of *PRKAR1A* and *PRKAR2A* observed in Cluster 9 may represent the initial event of reduced cAMP following CM-10–DOR interaction in the pathway.

The cAMP response element-binding protein (CREB), which is most likely localized in the nucleus, acts as a transcriptional regulator that binds to the cAMP response element (CRE) of the promoters of its target genes. In this study, *BRCA1*, *STAT3*, and *CREBBP*, which are transcriptional regulators of the cAMP response, were also downregulated in Cluster 10. Notably, regulatory proteins remain inactive, whereas phosphorylated proteins can bind to CRE and activate downstream gene expression [53,54]. *BRCA1* requires coactivator proteins and CREB-binding protein to activate target gene transcription [55]. Notably, the present study revealed the involvement of CREB in signaling pathways, indicating that it could be a potential target for treating mental disorders [53,56]. In addition to its role in neurodegenerative diseases, CREB may also be involved in the disease process of psychiatric disorders such as schizophrenia [57], autism [58], and depression [59].

A previous animal study reported an anxiolytic effect of orally administered CM- 10 in a plus maze test [21]. Moreover, a traditional Chinese medical compound with anxiolytic effects downregulated the NOTCH signaling pathway and promoted neuronal regeneration in the hippocampus of rats with generalized anxiety disorders [60]. *NOTCH1*, *NOTCH2*, and *ANK2* have also been associated with mental disorders, such as autism spectrum disorder [60,61,62,63,64]. Therefore, the downregulation of *NOTCH1* and *NOTCH2* following CM-10 treatment (Cluster 5 in Figure S1) could be a crucial event underlying the anxiolytic effect [60,61].

Interestingly, various genes involved in the circadian rhythm, such as *CLOCK*, *PER1*, *ARNTL*, and *RAI1*, were negatively regulated following CM-10 exposure (Cluster 6 in Figure S1). Notably, alterations in transcriptional rhythms have been associated with sleep and circadian traits (e.g., insomnia) [65], further supporting the biological relationships between changes in sleep, circadian rhythms, and synaptic signaling in opioid addiction [66,67,68,69,70,71,72]. The *ARNTL* gene product is a transcriptional activator that forms a core component of the circadian clock and plays a role in mood-related behavior [73]. Various genes involved in circadian rhythm processes are repressed by chronic morphine exposure in mice [74]. Furthermore, the agonistic opioid fentanyl induced phase shifts in circadian rhythms in hamsters [75]. This effect was explained by the direct action of fentanyl on the suprachiasmatic nuclei and regulation of *PER* genes [76,77]. Some opioid agonists affect the expression of specific genes involved in the circadian rhythm [78,79]. DOR agonists modulated light-induced phase advances in hamster circadian activity rhythms [78,79]. These reports support our data, suggesting that CM-10 may regulate circadian rhythms. Moreover, *TRAPPC* genes, including *TRAPPC11*, *TRAPPC10*, *TRAPPC9* [36], *TRAPPC8*, *TRAPPC6B*, and *TRAPPC2L*, which are involved in enteric neuronal differentiation associated with the downregulated activation of NF-κB, are also negatively regulated following CM-10 treatment (Cluster 3 in Figure S1).

This study had some limitations. First, we predicted networks based on transcriptome analysis of cultured DOR-HEK293 cells after treatment for a short time. However, culture cells often show different responses depending on the cell state and other conditions. Validation of the results using alternative cell models is required with triplicated samples. Second, some key gene products require evaluations using specific antibodies to clarify their critical changes. Third, exploring analogous agonist pathways in animal models following CM-10 treatment is crucial for validating our current findings. Further research focusing specifically on CM-10’s effects on anxiety and circadian rhythms in mice is particularly needed. In animal studies, mouse behavior within the circadian rhythm and related gene expression in the brain and gut should be verified. Therefore, comparative analysis between CM-10 and antagonists, along with demonstrating specific interactions with DOR, is essential for more detailed mechanistic analysis.

## 5. Conclusions

This study, through RNA-seq analysis, provided the first evidence of the broad impact of the opioid peptide CM-10 on gene expression in DOR-HEK293 cells. Transcriptome-wide network analysis indicated a tendency toward mild downregulation of gene expression in CM-10-treated DOR-HEK293 cells, particularly affecting regulatory subunits of cAMP-dependent protein kinases, cAMP response element-binding pathways, circadian rhythms, and genes associated with anxiety and depression. To clarify the underlaying mechanisms of CM-10 action, Supplementary Figure S1, including additional qPCR analysis, is required. In summary, our findings support the hypothesis that CM-10 exerts DOR agonist effects in DOR-HEK293 cells via a cAMP reduction mediated by specific signaling pathways.

## Figures and Tables

**Table 1:** Primer sequences for human clock genes used for reverse transcription-quantitative polymerase chain reaction.

Gene	Gene Name	Forward Primer Sequence	Reverse Primer Sequence
***PER1***	period circadian clock 1	5’-GCT CCT ACC AGC AGA TCA AC-3’	5’-GAG GCA CAT TTA CGC TTA GTG-3’
***PER2***	period circadian clock 2	5’-CAC AAA GCA AAA ATG AAC ACT G-3’	5’-CTC TCT GTC CTC CTT CAA AAC-3’
***CRY1***	cryptochrome circadian regulator 1	5’-CTC CGA TTT GGT TGT TTG TC-3’	5’-GAA AAA TTC ACG CCA TAA CAG-3’
***BMAL1***	basic helix-loop-helix ARNT like 1	5’-GAC AAC GAA CCA GAC AAT GAG-3’	5’-GTG CCG AGA AAC ATA TTC CAT AG-3’
***CREBBP***	CREB binding protein	5’-GAG ATG ATG GAG GAG GAT TTG-3’	5’-CGT TAC TGC TAC TCT CTT CTT C-3’
***ACTIN***	actin beta (ACTB), mRNA	5’-TGG CAC CCA GCA CAA TGA A-3’	5’-CTA AGT CAT AGT CCG CCT AGA AGC A-3’

**Table 2:** Gene clusters in down-regulated genes in DOR-HEK239 cells. Fourteen genes involved in circadian rhythm 275 were indicated in red.

Cluster	Genes	Gene Names in the Cluster	Function
**Cluster 1**	32	*AKAP11*, *ATAD2*, *C2CD3*, *CCDC14*, *CDK5R1*, *CDK5RAP2*, *CENPE*, *CEP135*, *CEP152*, *CEP295*, *CHML*, *CNTROB*, *DNAJC5*, *FGD6*, *IREB2*, *KIAA0753*, *KNL1*, *MINK1*, *NCKAP5L*, *NIN*, *ODF2*, *OFD1*, *PLK2*, *PLK3*, *RAB3A*, *SLC11A2*, *SLC38A1*, *SLC7A5*, *STX3*, *STXBP5*, *TFRC*, *ZRANB3*	Cell cycle regulation
**Cluster 2**	42	*ALG10*, *ALG10B*, *BMS1*, *CARMIL1*, *CCSER2*, *CD2AP*, *EDEM3*, *FAM160A2*, *FAM160B1*, *FTSJ3*, *HOOK1*, *INF2*, *ITPR3*, *NDEL1*, *NECTIN3*, *NLE1*, *NOL10*, *OBSCN*, *OGDH*, *PACS2*, *PAFAH1B2*, *PDCD11*, *PDCD6IP*, *PDK4*, *PPAN*, *PVR*, *RBM19*, *RRP1*, *RRP12*, *RRP1B*, *SHROOM3*, *STT3B*, *SUN1*, *SUN2*, *TBL3*, *TMEM201*, *TPM1*, *TPM4*, *TRMT2A*, *ULBP3*, *UTP15*, *UTP20*	Nuclear migration and matric anchoring, endonucleolytic cleavage
**Cluster 3**	30	*ASB7*, *CERS5*, *CERS6*, *COL4A1*, *CUL5*, *EXOC1*, *EXOC4*, *EXOC6*, *EXOC6B*, *GCC2*, *GOLGA4*, *ITGAV*, *ITGB8*, *MACF1*, *MAP11*, *PAQR3*, *RAB43*, *RHOBTB3*, *SGPL1*, *SMPD4*, *TENT4B*, *TMF1*, *TRAPP*, *TRAPPC10*, *TRAPPC11*, *TRAPPC2L*, *TRAPPC6B*, *TRAPPC8*, *TRAPPC9*, *TUT4*, *ZCCHC14*	Autophagy, Mental retardation
**Cluster 4**	20	*AQR*, *CCDC18*, *CCDC66*, *CCDC97*, *CTTNBP2NL*, *INTS1*, *INTS7*, *KIN*, *PCNX3*, *PPIL2*, *PPME1*, *PPP2R1B*, *PPP2R5E*, *PRPF3*, *PRPF4B*, *RBM41*, *SF3B1*, *SLMAP*, *SRRM2*, *STRN*	mRNA splicing, RNA processing
**Cluster 5**	42	*AMER1*, *ANK2*, *ARIH2*, *AXIN1*, *CANX*, *CELSR1*, *CHUK*, *CUL9*, *DLST*, *DVL1*, *FZD1*, *FZD5*, *GCDH*, *HIPK2*, *IKBKB*, *IL6ST*, *ITPR2*, *KITLG*, *MATK*, *NCSTN*, *NOTCH1*, *NOTCH2*, *PELI1*, *PLXNA1*, *PLXNB1*, *PLXNB2*, *PLXND1*, *PORCN*, *RFXAP*, *SEMA3F*, *SEMA4D*, *SEMA4G*, *SEMA6A*, *SFRP1*, *SPTB*, *SPTBN1*, *STIM1*, *TAB1*, *TAB3*, *TMOD2*, *WNT11*, *ZMYND11*	Semaphorin receptor complex, cellular response to tumor
**Cluster 6**	32	*AGO1*, *AGO2*, *AHR*, *ARNT2*, *ARNTL*, *ASXL3*, *CHD3*, *CHD6*, *CIPC*, *CLOCK*, *CNOT1*, *CNOT2*, *DAB2IP*, *DDX6*, *DGCR8*, *EPG5*, *EZH2*, *JARID2*, *KDM6A*, *KDM6B*, *MAU2*, *NR1D1*, *NR1D2*, *PDE12*, *PDS5A*, *PER1*, *RAI1*, *SIM2*, *SMARCC2*, *TET1*, *TXLNA*, *ZNF512B*	CLOC-BMAL regulation, Histone methylation
**Cluster 7**	41	*ARL4A*, *ATG9A*, *BRD1*, *BRPF3*, *BTAF1*, *CAPRIN1*, *CCPG1*, *EIF3B*, *EIF3C*, *EIF4G3*, *EPOR*, *INO80*, *KAT6A*, *LIFR*, *LSM12*, *MAPKAP1*, *MAX*, *MBTD1*, *MGA*, *MLST8*, *MXD4*, *PCF11*, *RB1CC1*, *RPRD1A*, *RPRD2*, *RPTOR*, *SEPTIN10*, *SEPTIN12*, *SLC30A7*, *SLC39A10*, *SLC39A6*, *SRCAP*, *STAM*, *STAM2*, *TAF2*, *TAF4B*, *TELO2*, *TRRAP*, *TTF2*, *TYK2*, *YEATS2*	Histone acetylation, TOR complex
**Cluster 8**	49	*ADGRB2*, *ARID1B*, *ARID2*, *BCL9*, *BCL9L*, *BICRA*, *BRD9*, *CDC73*, *DCAF16*, *DCAF4*, *DCAF8*, *EHD1*, *GAN*, *GATAD1*, *ICE1*, *KBTBD8*, *KLC3*, *KLC4*, *KLHL21*, *LZTR1*, *MAPK8IP3*, *NEMP1*, *NUP155*, *NUP210*, *NUP58*, *PHKA2*, *PHKG2*, *POM121*, *PYGB*, *PYGO1*, *PYGO2*, *RAB11FIP1*, *RAB11FIP2*, *RANGAP1*, *SENP5*, *SENP6*, *SIN3B*, *SMG5*, *SMG6*, *SMG7*, *SNAP47*, *TGFBRAP1*, *TLE4*, *TNRC18*, *USPL1*, *VPS18*, *VPS33A*, *VPS39*, *WDTC1*	RNA export from nucleus
**Cluster 9**	22	*AKAP10*, *CABYR*, *CLUAP1*, *DOP1A*, *GLI3*, *IFT81*, *MON2*, *MPP6*, *NCR3LG1*, *POLA1*, *PRIM2*, *PRKAR1A*, *PRKAR2A*, *TCTN2*, *TEN1*, *TMEM216*, *TMEM67*, *TRAF3IP1*, *TTC21B*, *TTC30A*, *TULP3*, *VPS26B*	DNA replication, cAMP—dependent kinase repression
**Cluster 10**	30	*BARD1*, *BRCA1*, *BRCC3*, *BRIP1*, *CDC27*, *CREBBP*, *ERBB2*, *ESPL1*, *FBH1*, *GEN1*, *GINS1*, *IFITM*	DNA repair and recombination

## Data Availability

The original contributions presented in the study are included in the article/Supplementary Material, further inquiries can be directed to the corresponding author.

## Supplementary Materials

The following supporting information can be downloaded at https://www.mdpi.com/article/10.3390/life15101636/s1, Table S1. Downregulated gene expressions in DOR-HEK293 cells by CM-10 exposure; Table S2. Upregulated gene expressions in DOR-HEK293 cells by CM-10 exposure; Table S3. Biological Process in GO analysis; Table S4. Cellular component in GO analysis; Table S5. Molecular Function in GO analysis; Figure S1. Major clusters in network of down-regulated genes in HEK 293 DOR by CM-10 treatment.
