# Supplementary material for: Casomorphine-10 (CM-10) Peptide Orchestrates Circadian and Neurodevelopmental Gene Clusters via δ-Opioid Receptor Signaling: Insights from Transcriptome Analysis with δ-Opioid Receptor-Expressing HEK293 Cells
Source: Life (Basel). 2025 Oct 20;15(10):1636. doi: 10.3390/life15101636 (PMC12565452; doi:10.3390/life15101636)
Supplement: Supplementary file 1 [file life-15-01636-s001.zip › CM-10 gene expression Supplementary Figure S1 revised.pptx]

## Slide 1
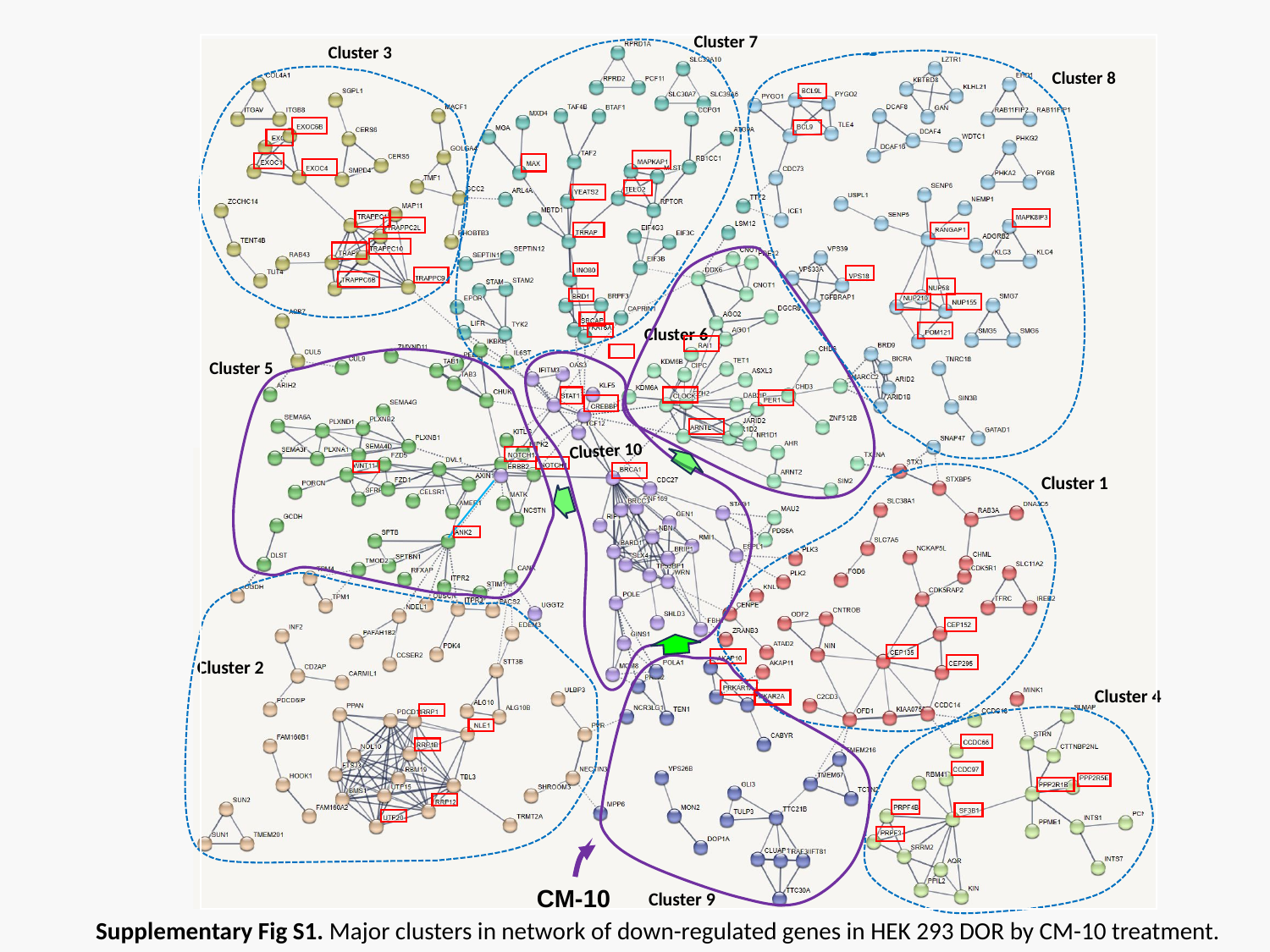

Cluster 7
Cluster 3
Cluster 8
Cluster 6
Cluster 5
Cluster 10
Cluster 9
Cluster 1
Cluster 2
Cluster 4
CM-10
Supplementary Fig S1. Major clusters in network of down-regulated genes in HEK 293 DOR by CM-10 treatment.
